# DOES THE SENSE OF MATTERING BUFFER DEPRESSIVE SYMPTOMS? EVIDENCE FROM THE CHAT PROGRAM SURVEY

**DOI:** 10.1093/geroni/igae098.3500

**Published:** 2024-12-31

**Authors:** Mengzhao Yan, Sheila Salinas Navarro, Sindy Lomeli, Kathleen Wilber

**Affiliations:** University of Southern California, Los Angeles, California, United States; University of Southern California, Los Angeles, California, United States; University of Southern California, Los Angeles, California, United States; University of Southern California, Los Angeles, California, United States

## Abstract

Sense of mattering refers to a feeling of being valued and important to others. Recognized as a unique construct and a form of psychological capital, mattering has profound implications on health and well-being. Previous research has demonstrated the protective effect of high level of mattering against mental health issues. However, this construct remains understudied in older adults. Employing the baseline survey (N = 2,385, average age = 71) of the “Connecting, Health, Aging, & Technology (CHAT) Program,” an initiative to bridge the digital divide for underserved community-dwelling older adults launched by the California Department of Aging, we examined the association between sense of mattering and depressive symptoms. Older adult participants identified by local area agencies on aging, were assessed and screened into the program. The relationship between participants’ scores on mattering (range 4 to 20) and having depressive symptoms was examined using multivariate logistic regression. After controlling for sociodemographic factors (i.e., age, gender, race/ethnicity, education, marital status), self-rated health, and anxiety, each unit higher level of mattering was associated with 12% lower odds of having depressive symptoms (OR = 0.88, 95% CI: 0.85 – 0.92). The five-item general mattering scale (Marcus, 1991) showed adequate internal consistency in older adults (Cronbach’s α = 0.80). Our findings highlight the importance of mattering to mental health. We recommend that future policies and programs aimed at improving older adults’ health and well-being (e.g., age-friendly movements, combating ageism, interventions) take boosting sense of mattering into account.

